# An Edge-Based Selection Method for Improving Regions-of-Interest Localizations Obtained Using Multiple Deep Learning Object-Detection Models in Breast Ultrasound Images

**DOI:** 10.3390/s22186721

**Published:** 2022-09-06

**Authors:** Mohammad I. Daoud, Aamer Al-Ali, Rami Alazrai, Mahasen S. Al-Najar, Baha A. Alsaify, Mostafa Z. Ali, Sahel Alouneh

**Affiliations:** 1 Department of Computer Engineering, German Jordanian University, Amman-Madaba Street, Amman 11180, Jordan; 2 Department of Diagnostic Radiology, The University of Jordan Hospital, Queen Rania Al-Abdullah Street, Amman 11942, Jordan; 3 Department of Network Engineering and Security, Jordan University of Science & Technology, Irbid 22110, Jordan; 4 Department of Computer Information Systems, Jordan University of Science & Technology, Irbid 22110, Jordan; 5 Cybersecurity Program, College of Engineering, Al Ain University, 28th Street, Abu Dhabi, United Arab Emirates

**Keywords:** medical ultrasound imaging, breast ultrasound images, computer-aided diagnosis, region-of-interest localization, deep learning object-detection models, deep learning edge-detection models

## Abstract

Computer-aided diagnosis (CAD) systems can be used to process breast ultrasound (BUS) images with the goal of enhancing the capability of diagnosing breast cancer. Many CAD systems operate by analyzing the region-of-interest (ROI) that contains the tumor in the BUS image using conventional texture-based classification models and deep learning-based classification models. Hence, the development of these systems requires automatic methods to localize the ROI that contains the tumor in the BUS image. Deep learning object-detection models can be used to localize the ROI that contains the tumor, but the ROI generated by one model might be better than the ROIs generated by other models. In this study, a new method, called the edge-based selection method, is proposed to analyze the ROIs generated by different deep learning object-detection models with the goal of selecting the ROI that improves the localization of the tumor region. The proposed method employs edge maps computed for BUS images using the recently introduced Dense Extreme Inception Network (DexiNed) deep learning edge-detection model. To the best of our knowledge, our study is the first study that has employed a deep learning edge-detection model to detect the tumor edges in BUS images. The proposed edge-based selection method is applied to analyze the ROIs generated by four deep learning object-detection models. The performance of the proposed edge-based selection method and the four deep learning object-detection models is evaluated using two BUS image datasets. The first dataset, which is used to perform cross-validation evaluation analysis, is a private dataset that includes 380 BUS images. The second dataset, which is used to perform generalization evaluation analysis, is a public dataset that includes 630 BUS images. For both the cross-validation evaluation analysis and the generalization evaluation analysis, the proposed method obtained the overall ROI detection rate, mean precision, mean recall, and mean F1-score values of 98%, 0.91, 0.90, and 0.90, respectively. Moreover, the results show that the proposed edge-based selection method outperformed the four deep learning object-detection models as well as three baseline-combining methods that can be used to combine the ROIs generated by the four deep learning object-detection models. These findings suggest the potential of employing our proposed method to analyze the ROIs generated using different deep learning object-detection models to select the ROI that improves the localization of the tumor region.

## 1. Introduction

Breast cancer is a major cause of deaths in females globally [1]. The International Agency for Research on Cancer reported 685,000 deaths around the globe due to breast cancer in 2020 [1]. Diagnosing breast cancer at early stages is important for achieving effective treatments and improving survival rates [2]. Many imaging modalities are used for diagnosing breast cancer, including mammography [3], ultrasound [4], magnetic resonance imaging [5], elastography [6], and microwave imaging [7]. Currently, mammography is the main imaging modality employed for diagnosing breast cancer [8]. Moreover, breast ultrasound (BUS) imaging is commonly used as an adjunct to mammography with the goal of enhancing the diagnosis process [9,10]. Despite the improved screening capabilities offered by BUS imaging, the interpretation of BUS images is challenging and requires experienced radiologists [11].

Computer-aided diagnosis (CAD) systems can be used to process BUS images and obtain computer-based classifications relative to the tumors [12]. The classifications generated by CAD systems provide radiologists with objective second opinions that aim to enhance the diagnosis process [13]. Many CAD systems, such as the systems introduced in [14,15], analyze the region-of-interest (ROI) that contains the tumor in the BUS image to extract handcrafted texture features for quantifying the tumor. The extracted texture features are analyzed using a computer classifier to classify the tumor as benign or malignant. Furthermore, many recent studies, such as [16,17,18], employed deep learning classification models to analyze the ROI that contains the tumor in the BUS image with the goal of achieving effective classifications of the tumor. Therefore, the development of fully automatic CAD systems requires the use of computer-based methods to localize ROIs that contain tumors in BUS images [19]. There are two main approaches for automatically identifying the ROI that contains the tumor in the BUS image. In the first approach, a segmentation algorithm is used to outline the tumor, and the detected tumor outline is employed to identify the ROI that contains the tumor [14]. In the second approach, the BUS image is analyzed directly using an ROI localization method to localize the ROI that contains the tumor [18]. The current study is focused on the field of developing automatic ROI localization methods that can directly analyze BUS images to localize ROIs that contain the tumors.

Several research groups proposed conventional methods to automatically localize ROIs that contain tumors in the BUS images. For instance, Shan et al. [20] introduced a two-step ROI localization method. In the first step, the texture and spatial characteristics of the BUS image are analyzed to localize a seed point inside the tumor region in the image. In the second step, the BUS image is processed using a region-growing algorithm to localize an ROI that contains a tumor based on the seed point obtained in the first step. However, the method introduced by Shan et al. [20] is characterized by limited localization accuracies when the tumor has low contrast compared to the surrounding tissues. Liu et al. [21] introduced an automated ROI localization method in which a texture-based support vector machine classifier was used to identify a group of candidate ROIs in the BUS image. The candidate ROIs are analyzed based on their vertical positions and their locations with respect to the image center to determine the final ROI that contains the tumor. The method introduced by Liu et al. [21] might have limited performance due to the non-robust criterion that was used to determine the final ROI. Xian et al. [19] proposed an adaptive four-step ROI localization method that aims to address the limitations of the method presented by Liu et al. [21]. In the first step, the BUS image is preprocessed to smooth the image and improve the appearance of hypoechoic regions. In the second step, the breast anatomy in the BUS image is analyzed to identify a reference point in the image. The reference point is processed using a multipath search algorithm to localize a seed point inside the tumor region. In the third step, the preprocessed BUS image was analyzed using a set of morphological operations followed by an adaptive thresholding algorithm to identify a group of candidate ROIs that are expected to include the tumor. In the fourth step, a selection algorithm is used to process the candidate ROIs based on the seed point to identify the final ROI that contains the tumor. The experimental results reported in [19] indicate that the method achieved low recall rates, which are equal to 27.7% and 30.9% for benign and malignant BUS images, respectively.

In the past few years, deep learning object-detection models, such as the models presented in [22,23,24,25,26,27], achieved great success in the general field of computer vision and image analysis. Nevertheless, a few research groups investigated the feasibility of applying deep learning object-detection models to localize ROIs in BUS images. For example, Cao et al. [18] evaluated the capability of five deep learning object-detection models to localize ROIs that contain the tumors in BUS images. These five models are the Single Shot MultiBox Detector (SSD) model [22], the Fast Region-Based Convolutional Neural Network (Fast R-CNN) model [23], the Faster Region-Based Convolutional Neural Network (Faster R-CNN) model [24], the You Only Look Once (YOLO) model [25], and the YOLO version 3 (YOLOv3) model [26]. In addition, Cao et al. [18] compared the performance of these five deep learning object-detection models with the conventional method introduced by Xian et al. [19]. The results reported in [18] show that the five deep learning object-detection models outperformed the conventional method introduced by Xian et al. [19]. This finding suggests the potential of using deep learning object-detection models to obtain the effective localization of ROIs in BUS images. Moreover, the results reported in [18] indicate that the SSD deep learning object detection model obtained higher ROI localization results compared with the other four deep learning object-detection models. In another study [28], the EfficientDet-D0 [29] and CenterNet [27] deep learning object-detection models have been compared in terms of their capability to localize ROIs that contain tumors in BUS images. The results reported in [28] indicate that the EfficientDet-D0 and CenterNet models achieved comparable ROI localization performance when they are trained using the same BUS image dataset. In a recent study, Yap et al. [30] presented a new approach for localizing the ROI that contains the tumor in the BUS image based on the Faster R-CNN model [24]. In this approach, the grayscale BUS image is preprocessed to obtain a sharpened BUS image and a contrast-enhanced BUS image. The original BUS image, the sharpened BUS image, and the contrast-enhanced BUS image are used as three RGB image channels to construct an artificial RGB image. The artificial RGB image is applied to the Faster R-CNN model to achieve an improved localization of the ROI that contains the tumor.

In the current study, a new selection method, called the edge-based selection method, was proposed to analyze ROIs generated by different deep learning object-detection models with the goal of selecting the ROI that improves the localization of the region that contains the tumor. The edge-based selection method is based on edge maps that are computed for the BUS images using the recently introduced Dense Extreme Inception Network (DexiNed) model [31]. In fact, the DexiNed model is a deep learning model that enables effective edge detection. To the best of our knowledge, our study is the first study that has employed a deep learning edge-detection model to detect the edges of tumors in BUS images. The proposed edge-based selection method is used to analyze the ROIs generated by four deep learning object-detection models, namely the Faster R-CNN [24], SSD [22], EfficientDet-D0 [29], and CenterNet [27] models, to select the ROI that provides an improved localization of the tumor region. Before employing the four deep learning object-detection models to localize the ROIs, BUS images are transformed into artificial RGB images to improve the capability of localizing the ROIs, as suggested in the study by Yap et al. [30]. The performance of the proposed edge-based selection method and the four deep learning object-detection models is evaluated using two BUS image datasets. The first dataset, named the JUH dataset, contains 380 BUS images. The second dataset, named the BUSI dataset, includes 630 BUS images. The results show that the proposed edge-based selection method has been able to improve ROI localizations generated using the four deep learning object-detection models in terms of ROI detection rates and the quality of the localized ROIs. In addition, the JUH dataset is used to compare the performance of the proposed edge-based selection method with three baseline-combining methods that can be employed to combine ROIs generated by the four deep learning object-detection models. These baseline-combining methods are the average-, union-, and intersection-based combining methods. The comparison results demonstrate the capability of the proposed edge-based selection method in outperforming the three baseline-combining methods. A major advantage of our proposed edge-based selection method is the capability to analyze ROIs generated using any group of deep learning object-detection models to select the ROI that improves the localization of the tumor region.

The remainder of the paper is organized as follows. Section 2 presents the proposed edge-based selection method. Additionally, Section 2 describes the use of the proposed edge-based selection method to analyze the ROIs generated by the Faster R-CNN, SSD, EfficientDet-D0, and CenterNet deep learning object-detection models. The experimental analyses employed to evaluate the performance of the proposed edge-based selection method as well as the four deep learning object-detection models are also provided in Section 2. Furthermore, Section 2 describes the analysis used to compare the proposed edge-based selection method with the three baseline-combining methods. Section 3, Section 4 and Section 5 present the results, discussion, and conclusion, respectively.

## 2. Methods and Materials

### 2.1. The Proposed Edge-Based Selection Method

For a given BUS image, different deep learning object-detection models can be used to generate ROIs that aim to localize the region that contains the tumor. The ROI generated by one deep learning object-detection model might be better than the ROIs generated by other models. In this study, an edge-based selection method was proposed with the goal of analyzing ROIs generated by different deep learning object-detection models to select the ROI that provides an improved localization of the region that contains the tumor.

The edge-based selection method is based on edge maps computed for the BUS images using the DexiNed deep learning edge-detection model [31]. The DexiNed model includes an encoder with 6 main blocks. The output of each main block is applied to an upsampling block to generate an intermediate edge map. The 6 intermediate edge maps were fused to generate the final edge map. A major advantage of the DexiNed model is the capability to train the model from scratch in an end-to-end learning manner to achieve effective edge detection. To the best of our knowledge, our study is the first study that has employed a deep learning edge-detection model to detect the edges of the tumors in BUS images. The process of training the DexiNed model to compute the edge maps of the BUS images is described in Section 2.3. The edge map computed by the DexiNed model is represented as a grayscale image with pixel intensities between 0 and 255. The pixels with intensity values close to 255 represent the edges of the tumor. Moreover, the pixels with intensity values close to 0 represent regions in the BUS image that do not include edges corresponding to the tumor. In the current study, the edge map computed using the DexiNed model is normalized by dividing the edge map by a value of 255. Hence, the pixels in the normalized edge map have intensity values between 0 and 1. For the sake of simplicity, we refer to the normalized edge map as the edge map in the rest of the paper.

**Figure 1:**
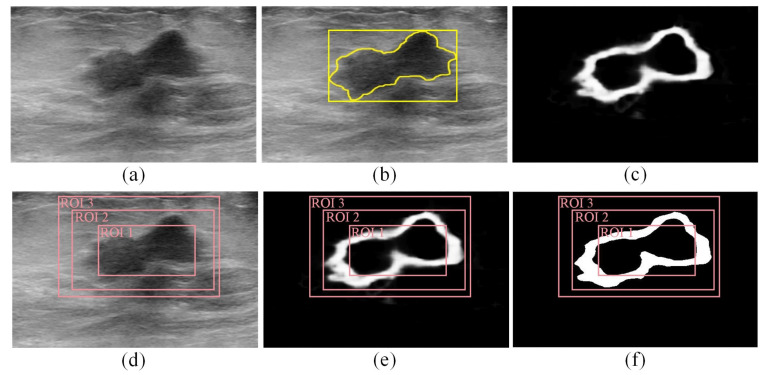
(**a**,**b**) (**a**) A malignant BUS image and (**b**) the corresponding gold standard tumor outline (yellow outline) and gold standard ROI (yellow box) that contains the tumor. (**c**) The edge map computed using the DexiNed model. (**d**–**f**) Three hypothetical ROIs (ROI 1, ROI 2, and ROI 3) overlaid on (**d**) the BUS image, (**e**) the edge map, and (**f**) the binarized edge map.

The edge map computed for a given BUS image using the DexiNed model can be used to analyze ROIs generated for that BUS image using different deep learning object-detection models. For example, consider the BUS image presented in Figure 1a. The gold standard tumor outline (yellow outline) and the gold standard ROI (yellow box) are shown in Figure 1b. The gold standard tumor outline is obtained by asking a radiologist (fourth author) with 15 years of experience in BUS imaging to segment tumor three times. The mean of the three manual segmentations is taken as the gold standard tumor outline. Furthermore, the gold standard ROI is taken as the smallest rectangular bounding box that contains the gold standard tumor outline. This approach for identifying the gold standard ROI has been employed in many previous studies, such as [18,30]. The edge map computed for the BUS image using the DexiNed model is presented in Figure 1c. Figure 1d,e show three hypothetical ROIs overlaid on the BUS image and the edge map, respectively. The first ROI, which is called ROI 1, covers a sub-region of the gold standard ROI and its area is smaller than the gold standard ROI by 50%. The second ROI, which is called ROI 2, covers the gold standard ROI, but its area is larger than the gold standard ROI by 25%. The third ROI, which is called ROI 3, covers the gold standard ROI, but its area is larger than the gold standard ROI by 75%. Hence, ROI 2 provides an improved localization of the region that contains the tumor compared to ROI 1 and ROI 3. Each one of the three hypothetical ROIs can be analyzed to select the ROI that provides an improved localization of the region that contains tumors.

The proposed edge-based selection method employs two indicators. The first indicator, denoted by *S*, is the sum of the edge map intensities of the pixels located within the ROI. The value of *S* can be computed as follows:

(1) $$S=\sum_{i=i_{s}}^{i_{e}}\sum_{j=j_{s}}^{j_{e}}EM\left(i,j\right)$$

where $i_{s}$ is the horizontal index of the pixels located at the left side of the ROI, $i_{e}$ is the horizontal index of the pixels located at the right side of the ROI, $j_{s}$ is the vertical index of the pixels located at the upper side of the ROI, $j_{e}$ is the vertical index of the pixels located at the lower side of the ROI, and EM(i,j) is the value of the edge map at the pixel with coordinates (i,j). The value of *S* increases when the ROI generated by the deep learning object-detection model enables the effective coverage of edges associated with the tumor’s boundary. Consequently, the value of *S* increases when the ROI includes a large number of true positive pixels, i.e., pixels that are located within both the gold standard ROI and the ROI generated by the deep learning object-detection model. For example, the values of *S* computed for ROI 1, ROI 2, and ROI 3 are equal to 5507, 12,683, and 12,780, respectively. Hence, ROI 1 has a value of *S* that is much smaller than the values of *S* computed for ROI 2 and ROI 3. Moreover, the value of *S* computed for ROI 2 is close, but smaller, than the value of *S* computed for ROI 3. Indicator *S* provides an effective metric to eliminate ROI 1 that does not provide effective coverage of the tumor boundaries. However, the indicator, *S*, does not provide a good metric for differentiating between ROI 2 and ROI 3.

The second indicator is the density of the edge map, denoted by *D*, and it represents the percentage of pixels within the ROI that has high edge-map intensity values with respect to the total number of pixels located within the ROI. The value of *D* is computed in two steps. In the first step, the edge map is binarized using the adaptive intermode-thresholding method [32]. The pixels in the binarized edge map that have values of 1 correspond to the pixels in the edge map with high-intensity values. Moreover, the pixels in the binarized edge map that have values of 0 correspond to the pixels in the edge map with low-intensity values. In the second step, the value of *D* is computed as follows:

(2) $$D=\frac{\sum_{i=i_{s}}^{i_{e}}\sum_{j=j_{s}}^{j_{e}}BEM\left(i,j\right)}{M\times N}$$

where BEM(i,j) is the value of the binarized edge map at the pixel with coordinates (i,j), *N* is the width of the ROI measured in pixels, and *M* is the height of the ROI measured in pixels. Hence, the value of *D* decreases when the ROI includes a large number of false-positive pixels. In fact, the false-positive pixels represent pixels that are located within the ROI generated by the deep learning object-detection model but that are not included in the gold standard ROI. For example, Figure 1f shows the binarized edge map computed for the edge map presented in Figure 1c along with three hypothetical ROIs. The values of *D* computed for ROI 1, ROI 2, and ROI 3 are equal to 0.25, 0.26, and 0.18, respectively. Therefore, the *D* indicator provides an effective metric for differentiating between ROI 2 and ROI 3, where both ROIs have close *S* values, but they are different in terms of the number of false-positive pixels.

The two indicators, *S* and *D*, are combined to obtain a single metric, SD=S×D, that can be used to select the ROI that improves the localization of the region that contains the tumor. In particular, the ROI with the highest SD value is selected since it maximizes the number of true-positive pixels and minimizes the number of false-positive pixels that are included within the selected ROI. For example, in Figure 1f, the SD values computed for ROI 1, ROI 2, and ROI 3 are equal to 1377, 3298, and 2300, respectively. Therefore, ROI 2 is selected as the ROI that improves the localization of the region that contains the tumor.

**Figure 2:**
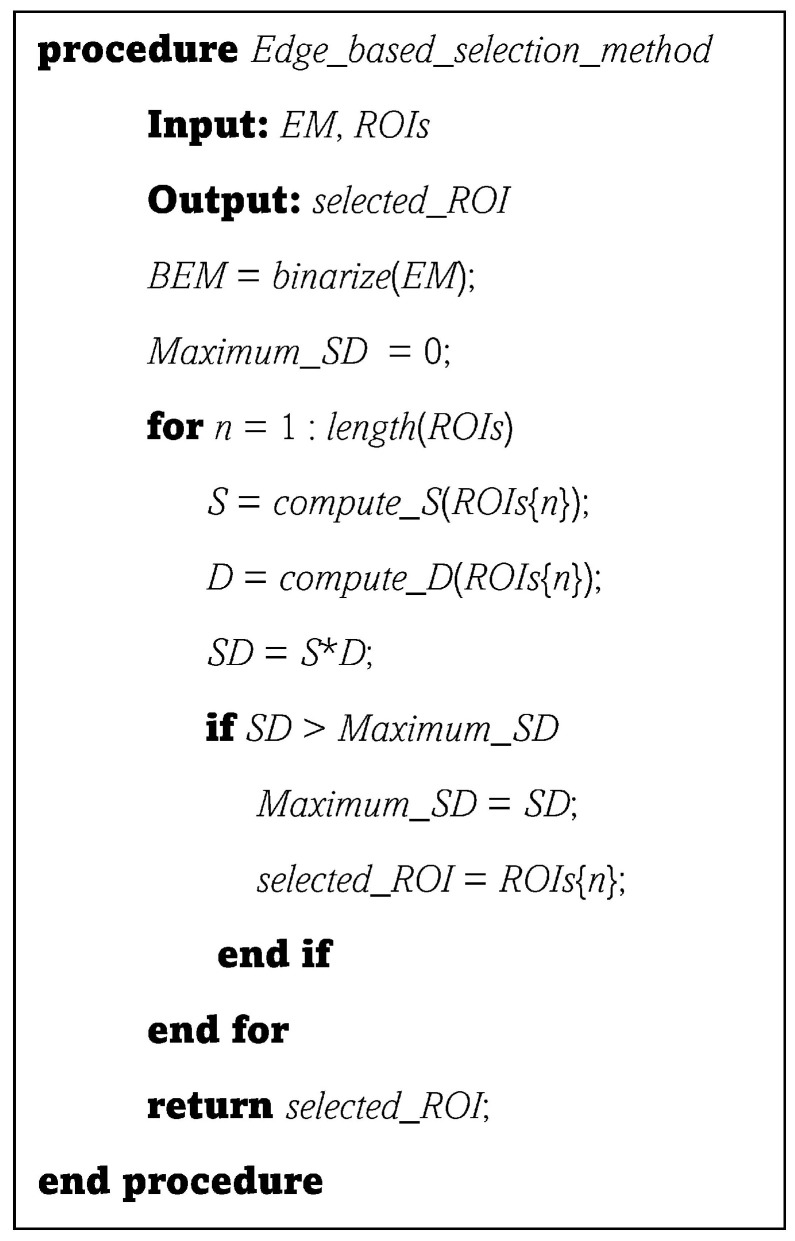
A procedure that summarizes the processes of employing the proposed edge-based selection method to analyze the ROIs generated by different deep learning object-detection models and select the ROI that enables the effective detection of the region that contains the tumor.

The procedure provided in Figure 2 describes the operation of the proposed edge-based selection method. The inputs of the procedure are the edge map, EM, of the BUS image and the set, ROIs, that includes the ROIs generated by the deep learning object-detection models. The output of the procedure is the ROI selected by the proposed edge-based selection method (selected_ROI). The functions employed by the procedure are the binarize(.) function that accepts the edge map (EM) and returns the binarized edge map, the length(.) function that accepts the set ROIs and returns the number of ROIs included in the set, the compute_S(.) function that accepts an ROI and returns the *S* value of the ROI, and the compute_D(.) function that accepts an ROI and returns the *D* value of the ROI.

### 2.2. Employing the Proposed Edge-Based Selection Method to Analyze the ROIs Generated by the Faster R-CNN, SSD, EfficientDet-D0, and  CenterNet Models and Select the ROI That Enables the Effective Detection of the Region That Contains the Tumor

**Figure 3:**
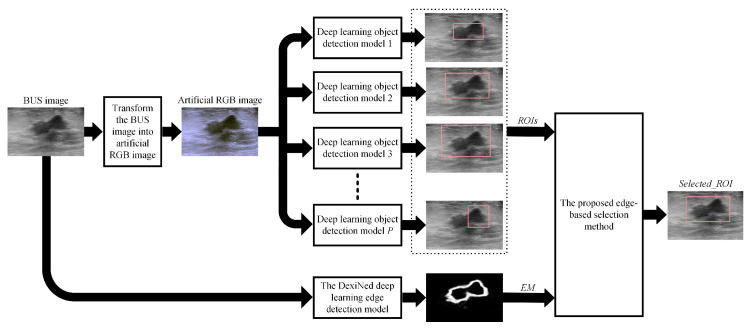
Graphical illustration of the process employed to apply the proposed edge-based selection method to analyze the ROIs generated by different deep learning object-detection models.

Figure 3 provides a graphical illustration of the process that we have employed to apply the proposed edge-based selection method to analyze ROIs generated by different deep learning object-detection models. At the beginning, the BUS image is transformed into artificial RGB images. The artificial RGB image is resized to match the input image size of the deep learning object-detection model, and the resized artificial RGB image is used as an input image for the deep learning object-detection model. In the current study, four deep learning object-detection models have been used, and these models include the Faster R-CNN, SSD, EfficientDet-D0, and CenterNet. The main similarities and differences between the four deep learning object-detection models are summarized in Table 1. Each deep learning object-detection model is used to localize the ROI that contains the tumor. Moreover, the ROIs generated by the different deep learning object-detection models are resized to match the size of the original BUS image. The resized ROIs are combined to synthesize the set of *ROIs*. In addition, the BUS image is processed using the DexiNed deep learning edge-detection model to obtain the edge map, which is called *EM*. The set, *ROIs*, and the edge map, *EM*, are applied to the proposed edge-based selection method to select the ROI, which is called *selected_ROI*, that provides an improved localization of the region that contains the tumor. The following subsections provide a detailed description of each component of this process.

#### 2.2.1. Transforming the BUS Images into Artificial RGB Images

The BUS image can be processed directly using the deep learning object-detection models to localize the ROI that contains the tumor. However, the study by Yap et al. [30] suggested that improved ROI localizations can be achieved by preprocessing the grayscale BUS image to generate two filtered images, namely a sharpened BUS image and a contrast-enhanced BUS image. The original BUS image and the two filtered images are used as three image channels to construct an artificial RGB image. The artificial RGB image can be processed by the deep learning object-detection models to achieve improved ROI localizations. In the current study, the procedure proposed by Yap et al. [30] was used to transform each BUS image into an artificial RGB image, and the artificial RGB image was employed as an input image for each one of the four deep-learning object detection models to localize the ROI that contains the tumor.

**Figure 4:**
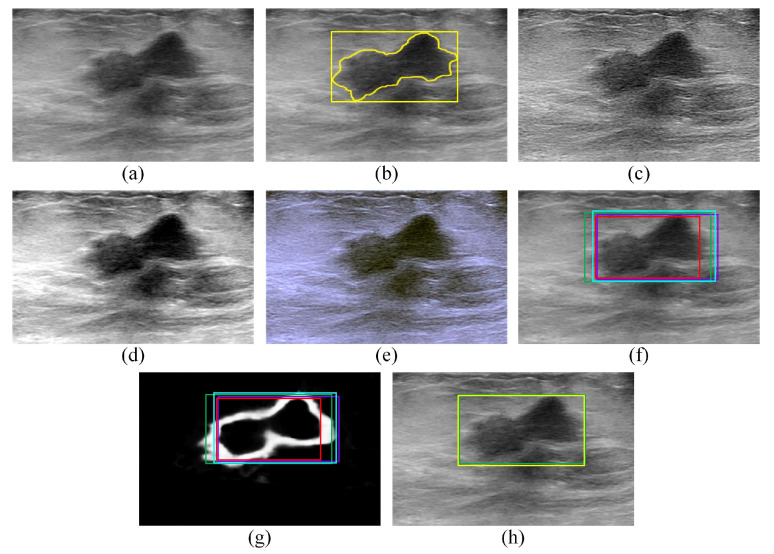
(**a**,**b**), the (**a**) A malignant BUS image and (**b**) the corresponding gold standard tumor outline (yellow outline) and gold standard ROI (yellow box) that contains the tumor. (**c**–**e**) The (**c**) sharpened image, (**d**) contrast-enhanced image, and (**e**) artificial RGB image computed for the B-mode image presented in (**a**). (**f**) The ROI localizations generated by the Faster R-CNN (violet box), SSD (red box), EfficientDet-D0 (cyan box), and CenterNet (green box) models overlaid on the BUS image. (**g**) The ROI localizations generated by the Faster R-CNN (violet box), SSD (red box), EfficientDet-D0 (cyan box), and CenterNet (green box) models overlaid on the edge map. (**h**) The ROI selected by the proposed edge-based selection method (in this case, the ROI generated by the CenterNet model—green box) as well as the gold standard tumor outline (yellow box) overlaid on the BUS image.

For instance, consider the malignant BUS image presented in Figure 4a, which is the same BUS image shown in Figure 1a. The gold standard tumor outline (yellow outline) and the gold standard ROI (yellow box) are presented in Figure 4b. Figure 4c,d show the sharpened image and contrast-enhanced image, respectively, that are computed for the BUS image in Figure 4a. Moreover, Figure 4e shows the artificial RGB image that is constructed by concatenating the BUS image, the sharpened image, and the contrast-enhanced image.

#### 2.2.2. Employing the Faster R-CNN Model to Localize ROIs That Contain the Tumors

The Faster R-CNN model [24] is a two-stage object-detection model that uses a pre-trained convolution neural network (CNN) to analyze the input image. Similarly to the study by Yap et al. [30], the model is configured to use the ResNet-50 CNN [33] that is pre-trained using the ImageNet database [34]. The last convolution layer of the ResNet-50 CNN is utilized to extract a deep feature map, which was employed to localize the tumor in the input image. The extracted deep feature map is processed in two stages. In the first stage, the region proposal network (RPN) generates rectangular region proposals, which represent potential regions in the input image that might contain the candidate object (i.e., the tumor). The generated region proposals have a predefined number as well as predefined sizes and aspect ratios. The RPN also computes an objectiveness score for each generated region proposal. Similarly to the study by Yap et al. [30], the RPN is configured to generate 100 region proposals. Moreover, the region proposals are set to have four different scales $(\frac{1}{4}$, $\frac{1}{2},1,2)$ and three different aspect ratios $\left(\frac{1}{2},1,2\right)$. In the second stage, the region proposals obtained by the RPN are refined using the Fast R-CNN detector [23]. In particular, the Fast R-CNN detector is used to process the region’s proposals as well as the feature map extracted using the ResNet-50 CNN. Each region proposal was employed to extract features from the feature map. The extracted features are processed using an ROI pooling layer to compute a fixed-size feature vector for each region proposal. The computed feature vectors are analyzed using a box classifier to classify and refine the region proposals. Using this process, two outputs are obtained. The first output is the likelihoods estimated for the object classes considered by the Faster R-CNN model, where one object class (i.e., the tumor) is employed in our study. The second output is the coordinates of the identified boxes. The box with the highest tumor likelihood is selected as the ROI that contains the tumor.

The Faster R-CNN model has been pre-trained using the MS-COCO dataset [35]. This dataset represents a large-scale image database for object detection that includes 380,000 images grouped into more than 90 object categories. The parameters used for pre-training the Faster R-CNN model are described in [24]. The process of fine-tuning the Faster R-CNN model to localize the ROIs that contain tumors in the artificial RGB images is described in Section 2.3.

For example, consider the BUS image and the artificial RGB image presented in Figure 4a,e, respectively. The ROI generated by the Faster R-CNN model is shown in Figure 4f,g as a violet box overlaid on the BUS image and the edge map, respectively.

#### 2.2.3. Employing the SSD Model to Localize the ROIs That Contain the Tumors

The SSD model [22] is a one-stage object-detection model that includes two main components: the base network and the auxiliary structure. The base network, which aims to extract deep feature maps from the input image, is realized using a pre-trained CNN that is truncated to eliminate classification layers. Similarly to the Faster R-CNN model, the SSD model is configured to use the ResNet-50 CNN, which is pre-trained using the ImageNet database as a base network. The auxiliary structure includes a group of multi-scale convolution layers that are appended to the base network. These convolution layers aimed to predict the target object (i.e., the tumor) at multiple scales. As suggested in the study by Miao et al. [36], six multi-scale convolution layers with linearly decreasing scales (between 0.95 and 0.2) have been used. Moreover, the model is configured to use three aspect ratios $\left(\frac{1}{2},1,2\right)$. The predictions obtained by the multi-scale convolution layers are processed using non-maximum suppression to obtain the final box that includes the target object. The final box is taken as the ROI that contains the tumor.

The SSD model has been pre-trained using the MS-COCO dataset. The parameters used for pre-training the SSD model are described in [22]. The process of fine-tuning the SSD model to localize the ROIs that contain the tumors in the artificial RGB images is described in Section 2.3.

For example, consider the BUS image and the artificial RGB image presented in Figure 4a,e, respectively. The ROI generated by the SSD model is shown in Figure 4f,g as a red box overlaid on the BUS image and the edge map, respectively.

#### 2.2.4. Employing the EfficientDet-D0 Model to Localize ROIs That Contain Tumors

The EffificientDet-D0 model [29] is a one-stage object-detection model that includes three main components: the backbone network, the weighted bidirectional feature pyramid network (BiFPN), and the class and box prediction network. The EfficientNet-B0 CNN [37], which is pretrained using the ImageNet database, is used as a backbone network. The aim of the EfficientNet-B0 CNN is to process the input image and compute the deep feature maps. The BiFPN extracts deep features from the backbone network and performs multi-scale weighted feature fusions along bottom-up and top-down directions. Finally, the class and box prediction network analyzes the fused features with the goal of predicting and localizing the target object (i.e., the tumor). The EffificientDet-D0 model is configured to use three aspect ratios $\left(\frac{1}{2},1,2\right)$. The other parameters of the EffificientDet-D0 model are set to their default values, as described in [29].

The EffificientDet-D0 model has been pre-trained using the MS-COCO dataset. The parameters used for pre-training the EffificientDet-D0 model are described in [29]. The process of fine-tuning the EffificientDet-D0 model to localize the ROIs that contain the tumors in the artificial RGB images is described in Section 2.3.

For example, consider the BUS image and the artificial RGB image presented in Figure 4a,e, respectively. The ROI generated by the EffificientDet-D0 model is shown in Figure 4f,g as a cyan box overlaid on the BUS image and the edge map, respectively.

#### 2.2.5. Employing the CenterNet Model to Localize ROIs That Contain Tumors

The CenterNet model [27] is a one-stage object-detection model that employs an anchor-free approach for object detection. In particular, the object-detection problem is expressed as keypoint localizations and bounding boxe regression problems [27]. The model processes the input image using a convolutional encoder–decoder backbone network to extract feature maps that support object detection. In this study, the backbone network is configured to use the ImageNet per-trained ResNet-50 CNN. The feature maps are processed using center pooling to compute a center heatmap, which aims to predict center keypoints for the target object. Moreover, the feature maps are processed using cascade corner pooling to compute corner heatmaps, which aim to estimate the corners of the potential bounding boxes that might contain the target object. The potential bounding boxes are analyzed based on the predicted center keypoints to identify a group of final bonding boxes that are expected to include the target object. The bounding box with the highest score is selected and used as the ROI that contains the tumor.

The CenterNet model is pre-trained using the MS-COCO dataset. The parameters used for pre-training the CenterNet model are described in [27]. The process of fine-tuning the CenterNet model to localize the ROIs that contain the tumors in the artificial RGB images is described in Section 2.3.

For example, consider the BUS image and the artificial RGB image presented in Figure 4a,e, respectively. The ROI generated by the CenterNet model is shown in Figure 4f,g as a green box overlaid on the BUS image and the edge map, respectively.

#### 2.2.6. Employing the Proposed Edge-Based Selection Method to Select ROIs That Enable the Effective Detection of Regions That Contain Tumors

The proposed edge-based selection method is used to process the four ROIs generated by the Faster R-CNN, SSD, EfficientDet-D0, and CenterNet models with the goal of selecting the ROI that improves the localization of the region that contains the tumor in the BUS image. For example, consider the ROIs in Figure 4f,g that are generated by the Faster R-CNN (violet box), SSD (red box), EfficientDet-D0 (cyan box), and CenterNet (green box) models. The SD values computed for the ROIs generated by the Faster R-CNN, SSD, EfficientDet-D0, and CenterNet models are equal to 2732, 2328, 3173, and 3605, respectively. The SD value calculated for the ROI generated by the CenterNet model is higher than the SD values computed for the ROIs generated by the three other models. Therefore, the ROI generated by the CenterNet model (green box) is selected by the proposed edge-based selection method, as shown in Figure 4h. To facilitate the comparison between the ROI selected by the proposed edge-based selection method and the gold standard ROI, Figure 4h also presents the gold standard ROI (yellow box).

### 2.3. Performance Evaluation

#### 2.3.1. The BUS Image Datasets

Two BUS image datasets have been used to perform experimental evaluations. The first dataset, called the University of Jordan Hospital (UJH) dataset, includes 163 malignant and 217 benign BUS images. Hence, the total number of BUS images included in the UJH dataset is 380 images. All images have been acquired for female patients at the UJH, Amman, Jordan. For each patient, one BUS image was included in the dataset. Each image contains one breast tumor. The Acuson S2000 ultrasound system (Siemens AG, Munich, Germany) was used to acquire the BUS images. The ultrasound system was equipped with a 14L5 transducer. The mean ± standard deviation size of the acquired BUS images was 479 ± 110 pixels along the axial dimension and 546 ± 60 pixels along the lateral dimension. The Institutional Review Board committee at the UJH has approved the study’s protocol. Moreover, signed informed consent forms of the study protocol were collected from all patients. BUS images were classified as benign or malignant using biopsy procedures. For each BUS image, the gold standard tumor outline and the gold standard ROI are obtained using the procedure described in Section 2.1.

The second dataset is a public dataset, called the BUSI dataset [38], that includes 210 malignant and 437 benign BUS images. Hence, the total number of BUS images included in the BUSI dataset is 647 images. The dataset has been acquired in 2018 at the Baheya Hospital for Early Detection and Treatment of Women’s Cancer, Cairo, Egypt. Ultrasound imaging was performed using the LOGIQ E9 and LOGIQ E9 Agile systems (GE Healthcare Inc., Chicago, IL, USA). The ultrasound systems were equipped with an ML6-15-D Matrix transducer. The mean ± standard deviation size of the acquired BUS images was 495 ± 73 pixels along the axial dimension and 609 ± 120 pixels along the lateral dimension. The gold standard outlines of the BUS images, which are provided in the dataset, are drawn by an experienced radiologist. In the current study, the ROI localization process is performed under the assumption that the BUS image includes one tumor. However, the BUSI dataset contains 17 BUS images with multiple tumors. These 17 BUS images have been removed. Hence, the final dataset that is used in the current study is composed of 630 BUS images (209 malignant and 421 benign). The gold standard ROIs of the BUS images included in the BUSI dataset have been determined using the same procedure that was employed for the UJH dataset.

#### 2.3.2. Evaluating the Performance of the Proposed Edge-Based Selection Method and the Four Deep Learning Object Models Using the UJH Dataset

The UJH dataset is used to fine-tune and test the Faster R-CNN, SSD, EfficientDet-D0, and CenterNet deep learning object-detection models. Fine-tuning and testing processes are conducted using a ten-fold cross-validation procedure. In this cross-validation procedure, the 380 artificial RGB images, which correspond to the 380 BUS images included in the UJH dataset, are divided randomly into ten image subsets of equal sizes. Hence, each subset includes 38 artificial RGB images. In the first fold, the images included in the first subset are used as testing images and the remaining nine image subsets are used for training the models. In the second fold, the second subset is used for testing, and the remaining subsets are used for training. This process was repeated ten-fold to ensure that the images included in the ten subsets are used as testing images. The training of the four deep learning object-detection models has been carried out using the stochastic gradient descent optimizer [39], a learning rate of 0.01, and a number of epochs equals 100. In addition, the UJH dataset was employed to train and test the DexiNed deep learning edge-detection model, which is utilized by the proposed edge-based selection method to compute the edge maps of the BUS images. The training and testing of the DexiNed model is carried out using the ten-fold cross-validation procedure described above. The training of the DexiNed model is performed using the stochastic gradient descent optimizer, a learning rate of 0.0001, and a number of epochs equals 100.

The performance of the Faster R-CNN model, the SSD model, the EfficientDet-D0 model, the CenterNet model, and the proposed edge-based selection method is assessed in terms of the ROI detection rate and the quality of the localized ROIs. The ROI detection rate is defined as the number of images in which the ROI localization technique generates an ROI localization that overlaps partially or completely with the corresponding gold standard ROI, divided by the total number of images. The quality of the localized ROIs is evaluated using three metrics, which are the precision, recall, and F1-score. For a given BUS image, the three metrics can be computed as follows [18]:

(3) $$\begin{array}{c} \mathrm{Precision}=\frac{|{\mathrm{ROI}}^{\mathrm{GS}}\cap {\mathrm{ROI}}^{\mathrm{Pred}}|}{|{\mathrm{ROI}}^{\mathrm{Pred}}|}\\ \mathrm{Recall}=\frac{|{\mathrm{ROI}}^{\mathrm{GS}}\cap {\mathrm{ROI}}^{\mathrm{Pred}}|}{|{\mathrm{ROI}}^{\mathrm{GS}}|}\\ F1\text{-}\mathrm{score}=\frac{2\times \mathrm{Precision}\times \mathrm{Recall}}{\mathrm{Precision}+\mathrm{Recall}} \end{array}$$

where ${\mathrm{ROI}}^{\mathrm{GS}}$ is gold standard ROI, ${\mathrm{ROI}}^{\mathrm{Pred}}$ is the predicted ROI, $|{\mathrm{ROI}}^{\mathrm{GS}}\cap {\mathrm{ROI}}^{\mathrm{Pred}}|$ is the number of pixels within the overlap between ${\mathrm{ROI}}^{\mathrm{GS}}$ and ${\mathrm{ROI}}^{\mathrm{Pred}}$, $|{\mathrm{ROI}}^{\mathrm{Pred}}|$ is the number of pixels in ${\mathrm{ROI}}^{\mathrm{Pred}}$, and $|{\mathrm{ROI}}^{\mathrm{GS}}|$ is the number of pixels in ${\mathrm{ROI}}^{\mathrm{GS}}$. It is worth noting that the ROIs generated by the four deep learning object-detection models are resized to match the sizes of the corresponding gold standard ROIs, as explained in Section 2.2. The mean ± standard deviation values of the precision, recall, and F1-score are computed for the proposed edge-based selection method as well as the four deep learning object-detection models. In particular, for each ROI localization approach, the mean ± standard deviation values of the precision, recall, and F1-score metrics are computed for the benign, malignant, and all BUS images in which the ROI localization approach has successfully generated ROIs that overlap partially or completely with the corresponding gold standard ROIs.

#### 2.3.3. Comparing the Performance of the Proposed Edge-Based Selection Method with Other Baseline-Combining Methods Using the UJH Dataset

The results of the proposed edge-based selection method, which have been achieved using the UJH dataset, are compared with three baseline methods that can be used to combine the ROIs generated by the four deep learning object-detection models. These baseline methods are the average-based combining method, the union-based combining method, and the intersection-based combining method. The average-based combining method computes the average of the four ROIs generated by the four deep learning object-detection models. In particular, the upper-left corner of the average ROI corresponds to the center point of the upper-left corners of the ROIs generated by the Faster R-CNN, SSD, EfficientDet-D0, and CenterNet models. Moreover, the lower-right corner of the average ROI corresponds to the center point of the lower-right corners of the ROIs generated by the Faster R-CNN, SSD, EfficientDet-D0, and CenterNet models. Additionally, the union-based combining method computes the union of the four ROIs generated by the four deep learning object-detection models. Moreover, the intersection-based combining method computes the intersection of the four ROIs generated by the four deep learning object-detection models.

The performance of the average-, union-, and intersection-based combining methods is assessed using the UJH dataset by computing the ROI detection rates and the mean ± standard deviation values of the precision, recall, and F1-score metrics. For the three combining methods, if one or more of the deep learning object-detection models fails to generate an ROI for a given BUS image, then the failed models are excluded from the process of computing the combined ROIs. The ROI detection rates and the mean ± standard deviation values achieved by the average-, union-, and intersection-based combining methods are compared with the matching values obtained by the proposed edge-based selection method.

#### 2.3.4. Evaluating the Generalization Performance of the Proposed Edge-Based Selection Method and Four Deep Learning Object Models Using the BUSI Dataset

The generalization performance of the proposed edge-based selection method and the four deep learning object-detection models is evaluated using the BUSI dataset. In particular, the 380 artificial RGB images, which correspond to the 380 BUS images included in the UJH dataset, are used to fine-tune the four deep learning object-detection models and to train the DexiNed deep learning edge-detection model. The training parameters are set to match the parameters employed in the ten-fold cross-validation procedure described in Section 2.3.2. The fine-tuned Faster R-CNN, SSD, EfficientDet-D0, and CenterNet object-detection models are employed to localize the ROIs in the 630 artificial RGB images that correspond to the 630 BUS images included in the BUSI dataset. Furthermore, the trained DexiNed edge-detection model is used to compute the edge maps of the BUS images included in the BUSI dataset. The computed edge maps are employed to apply the proposed edge-based selection method to improve the ROI localizations in the BUS images included in the BUSI dataset. The performance of the proposed edge-based selection method, the Faster R-CNN model, the SSD model, the EfficientDet-D0 model, and the CenterNet model is assessed by computing the ROI detection rate and the mean ± standard deviation values of the precision, recall, and F1-score metrics. Particularly, for each ROI localization approach, the mean ± standard deviation values of the precision, recall, and F1-score metrics are calculated for benign, malignant, and all BUS images in which the ROI localization approach successfully generated ROIs that overlap partially or completely with the corresponding gold standard ROIs.

## 3. Results

### 3.1. The Results Obtained by the Proposed Edge-Based Selection Method and the Four Deep Learning Object Models Using the UJH Dataset

Table 2 shows the ROI detection rates obtained for benign, malignant, and all BUS images included in the UJH dataset using the Faster R-CNN, SSD, EfficientDet-D0, and CenterNet object-detection models as well as the proposed edge-based selection method. The ROI detection rates obtained using the EfficientDet-D0 object detection model for benign BUS images, malignant BUS images, and all BUS images are higher than the ROI detection rates achieved using the three other deep learning object-detection models. Furthermore, the ROI detection rates achieved using the proposed edge-based selection method are higher than the ROI detection rates obtained using the four deep learning object-detection models. Table 2 also shows the mean ± standard deviation precision, recall, and F1-score values obtained for the UJH dataset using the Faster R-CNN, SSD, EfficientDet-D0, and CenterNet object-detection models as well as the proposed edge-based selection method. The mean precision, recall, and F1-score values achieved using the CenterNet model for the benign BUS images, malignant BUS images, and all BUS images are higher than the matching mean precision, recall, and F1-score values obtained using the three other deep learning object-detection models. Additionally, the proposed edge-based selection method obtained mean precision, recall, and F1-score values for the benign BUS images, malignant BUS images, and all BUS images that are higher than the matching mean precision, recall, and F1-score values achieved using the four deep learning object-detection models. These results demonstrate that the proposed edge-based selection method is able to improve ROI localizations obtained using the four deep learning object-detection models.

**Figure 5:**
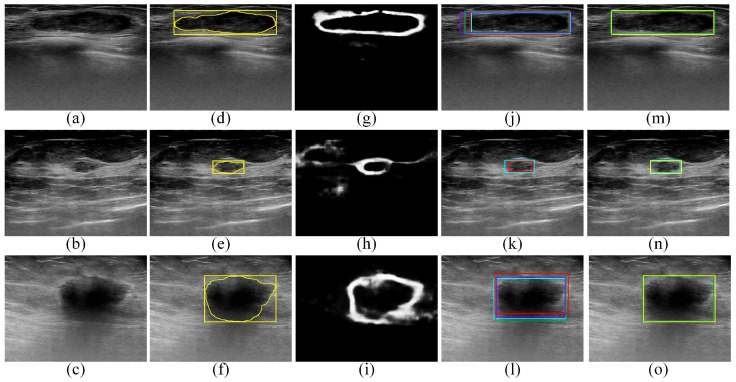
Qualitative results obtained for the UJH dataset. (**a**–**c**) BUS images acquired for (**a**) benign tumor and (**b**,**c**) malignant tumors. (**d**–**f**) The gold standard tumor outlines (yellow outlines) and the gold standard ROIs (yellow boxes) overlaid on the BUS images. (**g**–**i**) The edge maps computed using the DexiNed model. (**j**–**l**) The ROI localizations generated by the Faster R-CNN (violet boxes), SSD (red boxes), EfficientDet-D0 (cyan boxes), and CenterNet (green boxes) models overlaid on the BUS images. In (**k**), the Faster R-CNN and CenterNet models failed to generate ROIs. (**m**–**o**) The ROIs selected by the proposed edge-based selection method as well as the gold standard ROIs (yellow boxes) overlaid on the BUS images. In (**m**–**o**) the proposed edge-based selection method selected the ROIs generated by the CenterNet, EfficientDet-D0, and CenterNet models, respectively.

Figure 5 provides qualitative ROI localization results obtained for the UJH dataset by the Faster R-CNN, SSD, EfficientDet-D0, and CenterNet object-detection models as well as the proposed edge-based selection method. The qualitative results provided in Figure 5 demonstrate that the proposed edge-based selection method was able to process the ROIs generated by the four deep learning object-detection models to select the ROI that improves the localization of the region that contains the tumor.

### 3.2. The Results of Comparing the Proposed Edge-Based Selection Method with Other Baseline-Combining Methods Using the UJH Dataset

Table 3 shows the ROI detection rates and the mean ± standard deviation values of the precision, recall, and F1-score metrics that are obtained based on the UJH dataset using the average-, union-, and intersection-based combining methods as well as the proposed edge-based selection method. The average-, union-, and intersection-based combining methods obtained higher ROI detection rates than the ROI detection rates of the four individual deep learning object detection methods, which are presented in Table 2. Moreover, the union-based combining method achieved ROI detection rates that are equal to the proposed edge-based selection method and slightly higher than the average-based combining method and the intersection-based combining method.

The intersection-based combining method obtained mean precision values that are higher than the average-based combining method, the union-based combining method, and the proposed edge-based selection method. However, the intersection-based combining method achieved mean recall values that are substantially lower than the average-based combining method, the union-based combining method, and the proposed edge-based selection method. The union-based combining method obtained mean recall values that are higher than the average-based combining method, the intersection-based combining method, and the proposed edge-based selection method. However, the union-based combining method achieved mean precision values that are substantially lower than the than the average-based combining method, the union-based combining method, and the proposed edge-based selection method. The average-based combining method obtained precision and recall values that are lower than the proposed edge-based selection method.

The F1-score metric, which combines the precision and recall metrics, can be used as a unified metric to evaluate the overall quality of the ROIs obtained using the three combining methods as well as the proposed edge-based selection method. Table 3 shows that the proposed edge-based selection method obtained F1-score values that are higher than the three baseline-combining methods. Moreover, Table 3 indicates that the average-based combining method achieved F1-score values that outperform the two other baseline-combining methods. In addition, the three baseline-combining methods obtained F1-score values that are lower than the F1-score values achieved by the CenterNet model, which are shown in Table 2. The results provided in Table 3 indicate that the proposed edge-based selection method outperformed the three baseline-combining methods.

### 3.3. The Generalization Results Obtained by the Proposed Edge-Based Selection Method and the Four Deep Learning Object Models Using the BUSI Dataset

Table 4 presents the ROI detection rates and the mean ± standard deviation values of the precision, recall, and F1-score that are obtained for the BUSI dataset using the four deep learning object-detection models as well as the proposed edge-based selection method. The EfficientDet-D0 object-detection model achieved ROI detection rates that are higher than the matching ROI detection rates obtained using the three other deep learning object-detection models. Additionally, the ROI detection rates achieved using the proposed edge-based selection method are higher than the matching ROI detection rates obtained using the four deep learning object-detection models. The mean precision, recall, and F1-score values obtained using the CenterNet model are higher than the matching mean precision, recall, and F1-score values obtained using the three other deep learning object-detection models. Furthermore, the proposed edge-based selection method achieved mean precision, recall, and F1-score values that are higher than the matching mean precision, recall, and F1-score values obtained using the four deep learning object-detection models. These results indicate that the ability of the proposed edge-based selection method to improve ROI localizations obtained using the four deep learning object-detection models can be generalized to other BUS image datasets.

**Figure 6:**
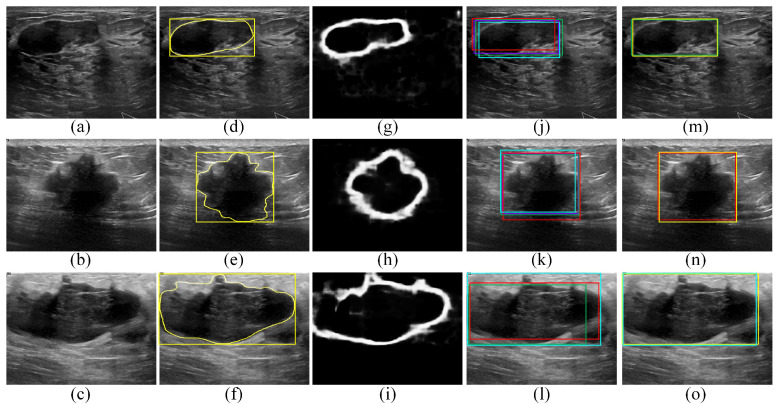
Qualitative results obtained for the BUSI dataset. (**a**–**c**) BUS images acquired for (**a**) benign tumor and (**b**,**c**) malignant tumors. (**d**–**f**) The gold standard tumor outlines (yellow outlines) and the gold standard ROIs (yellow boxes) overlaid on the BUS images. (**g**–**i**) The edge maps computed using the DexiNed model. (**j**–**l**) The ROI localizations generated by the Faster R-CNN (violet boxes), SSD (red boxes), EfficientDet-D0 (cyan boxes), and CenterNet (green boxes) models overlaid on the BUS images. In (**l**), the Faster R-CNN model failed to generate an ROI. (**m**–**o**) The ROIs selected by the proposed edge-based selection method as well as the gold standard ROIs (yellow boxes) overlaid on the BUS images. In (**m**–**o**) the proposed edge-based selection method selected the ROIs generated by the CenterNet, SSD, and EfficientDet-D0 models, respectively.

Figure 6 presents qualitative ROI localization results obtained for the BUSI dataset using the four deep learning object-detection models as well as the proposed edge-based selection method. The qualitative results provided in Figure 6 demonstrate the capability of the proposed edge-based selection method to process the ROI localizations generated by the four deep learning object-detection models and select an ROI that improves the localization of the region that includes the tumor.

## 4. Discussion

In this study, we have compared the capabilities of four deep learning object-detection models, namely the Faster R-CNN [24], SSD [22], EfficientDet-D0 [29] , and CenterNet [27], to localize the ROIs that include the tumors in BUS images. Our study also presents a new edge-based selection method that aims to analyze the ROI localizations generated by the four deep learning object-detection models and select ROIs that improve the localizations of tumors. The process of analyzing and selecting the ROIs is performed using edge maps that are computed for the BUS images using the DexiNed deep learning edge-detection model. In addition, the performance of the proposed edge-based selection method was compared with three baseline-combining methods, namely the average-, union-, and intersection-based combining methods.

Table 2 presents the ROI localization results obtained by the four deep learning object-detection models and the proposed edge-based selection method, based on the UJH dataset and using the ten-fold cross-validation procedure. The results show that the four object-detection models have different ROI localization capabilities in terms of the ROI detection rates and the quality of the localized ROIs. The EfficientDet-D0 model achieved the highest ROI detection rates, which are equal to 91% for the benign BUS images, 86% for the malignant BUS images, and 89% for all BUS images, compared to the three other deep learning object-detection models. Furthermore, the CenterNet model obtained the highest quality of the localized ROIs with mean precision, recall, and F1-score values equal to 0.89, 0.88, and 0.88, respectively, for the benign BUS images; 0.87, 0.86, and 0.85, respectively, for the malignant BUS images; and 0.88, 0.87, and 0.86, respectively, for all BUS images. Table 2 also shows that the proposed edge-based selection method is able to improve the detection rates and the quality of the ROIs generated by the four deep learning object-detection models. In particular, the ROI detection rates achieved using the proposed edge-based selection method are equal to 98% for the benign BUS images, 97% for the malignant BUS images, and 98% for all BUS images. The mean precision, recall, and F1-score values achieved using the proposed edge-based selection method are equal to 0.92, 0.91, and 0.91, respectively, for the benign BUS images; 0.89, 0.89, and 0.89, respectively, for the malignant BUS images; and 0.91, 0.90, and 0.90, respectively, for all BUS images.

Table 3 presents comparison results between the proposed edge-based selection method and the average-, union-, and intersection-based baseline-combining methods that are obtained using the UJH dataset. Similarly to the proposed edge-based selection method, the three baseline-combining methods have been able to achieve ROI detection rates that are higher than the individual deep learning object-detection models. The F1-score metric, which combines the precision and recall metrics, can be used as a unified metric to evaluate the quality of the ROIs obtained by the proposed edge-based selection method and the three baseline-combining methods. The results provided in Table 3 show that the proposed edge-based selection method achieved F1-score values that are higher than the three baseline-combining methods, which demonstrates the superior performance of the proposed method. Furthermore, the average-, union-, and intersection-based combining methods obtained F1-score values that are lower than the values achieved by the CenterNet deep learning object-detection model, which are presented in Table 2. These results indicate that the quality of the ROIs obtained by three baseline-combining methods is lower than the ROIs generated by the CenterNet model. Table 3 also shows that the union-based combining method achieved recall values that are slightly higher than the values obtained by the proposed edge-based selection method. This can be attributed to the fact that the ROI obtained by union-based combining method represents the union of all ROIs generated by the four individual deep learning object-detection models. Hence, the union ROI is expected to have high overlap with the gold standard ROI, which leads to high recall values. On the other hand, the union ROI is expected have a high number of false positive pixels, which leads to small precision values, as shown in Table 3. Furthermore, the results provided in Table 3 show that the intersection-based combining method obtained precision values that are higher than the values achieved by the proposed edge-based selection method. This can be attributed to the fact that the ROI obtained by intersection-based combining method represents the intersection between all ROIs generated by the four individual deep learning object-detection models. The intersection ROI is expected to be composed mainly of true positive pixels with a limited number of false positive pixels, which leads to high precision values. On the other hand, the ROI localization obtained by the intersection-based combining method is expected to have a high number of false negative pixels, which leads to small recall values, as shown in Table 3.

Table 4 shows the generalization results of the four deep learning object-detection models and the proposed edge-based selection method that are obtained by employing the UJH dataset for training and the BUSI dataset for testing. Among the four deep learning object-detection models, the EfficientDet-D0 model achieved the highest ROI detection rates, which are equal to 87% for the benign BUS images, 83% for the malignant BUS images, and 86% for all BUS images. Additionally, the CenterNet model achieved the highest ROI localization quality with mean precision, recall, and F1-score values equal to 0.88, 0.87, and 0.85, respectively, for the benign BUS images; 0.85, 0.84, and 0.83, respectively, for the malignant BUS images; and 0.87, 0.86, and 0.85, respectively, for all BUS images. Table 4 also shows that the proposed edge-based selection method has improved the detection rates and the quality of the ROI localizations obtained by the four deep learning object-detection models. In particular, the ROI detection rates achieved by the proposed edge-based selection method are equal to 99% for the benign BUS images, 96% for the malignant BUS images, and 98% for all BUS images. The mean precision, recall, and F1-score values obtained by the proposed edge-based selection method are equal to 0.91, 0.90, and 0.89, respectively, for the benign BUS images; 0.87, 0.88, and 0.86, respectively, for the malignant BUS images; and 0.90, 0.89, and 0.88, respectively, for all BUS images. These findings agree with the results provided in Table 2, which are obtained for the UJH dataset using the ten-fold cross-validation procedure.

A crucial factor that enables the proposed edge-based selection method to analyze ROI localizations generated by the four deep learning object-detection models and select the ROI that improves the localization of the tumor region is the use of the DexiNed deep learning edge-detection model to compute the edge map of the BUS image. To the best of our knowledge, our study is the first study that has used deep learning technology to detect the edges of tumors in BUS images. However, if the four deep learning object-detection models fail to generate ROIs that can be used to identify the region that contains the tumor, then the proposed edge-based selection method will select one of these ROIs without being able to identify the tumor region. To address this limitation, additional deep learning object-detection models can be employed to generate a larger number of candidate ROIs, and the proposed edge-based selection method can be used to process these ROIs to select the ROI that improves the localization of the tumor region. In fact, one advantage of the proposed edge-based selection method is the capability to analyze the ROIs generated by any group of object-detection models, without any restriction on the number of object-detection models that are used to generate the ROIs.

In future, we are planning to extend our study by employing the improved ROI localizations obtained by our proposed edge-based selection method to classify the tumors in BUS images. In particular, the improved ROI localizations obtained by our proposed edge-based selection method can be analyzed using deep learning BUS image-classification methods, such as the method introduced in [17], to achieve fully automated classification of breast tumors. The simple formulation of the proposed edge-based selection method, which is focused on maximizing the capability of containing the edges associated with the tumor in the ROIs and minimizing the number of false positive pixels, enables the expansion of our method to improve the ROI localization in other types of medical breast images, such as mammography, magnetic resonance imaging, and computed tomography images.

## 5. Conclusions

In this study, a new edge-based selection method is proposed to analyze the ROIs generated by different deep learning object-detection models with the goal of improving the localization of the ROIs that contain the tumors. The proposed method is based on edge maps computed for the BUS images using the recently introduced DexiNed deep learning edge-detection model. To the best of our knowledge, our study is the first study that has employed a deep learning edge-detection model to detect the edges of tumors in BUS images. Our proposed edge-based selection method has been employed to analyze the ROI localizations generated by four deep learning object-detection models, namely the Faster R-CNN, SSD, EfficientDet-D0, and CenterNet, and for selecting the ROI that improves the localization of the tumor region. The performance of the proposed edge-based selection method as well as the four deep learning object-detection models has been assessed in terms of the ROI detection rate as well as the precision, recall, and F1-score. The results, which are obtained using the UJH and BUSI datasets, indicate that our proposed edge-based selection method has been able to achieve ROI detection rates that are higher than the four deep learning object-detection models. Moreover, the results indicate that the mean precision, recall, and F1-score values achieved using our proposed edge-based selection method are higher than the matching values obtained by the four deep learning object-detection models. In addition, the performance of the proposed edge-based selection method has been compared with three baseline-combining methods, namely the average-, union-, and intersection-based combining methods, using the UJH dataset. The results show that our proposed edge-based selection method has been able to outperform the three baseline-combining methods. In future, we are planning to expand our proposed edge-based selection method by selection the ROIs generated by a larger group of deep learning object-detection models to enhance the capability of localizing ROIs that contain tumors in BUS images. Furthermore, we are planning to analyze the ROIs obtained by our proposed method to classify the tumors in BUS images using deep learning technology.

## Figures and Tables

**Table 1:** Comparison of the four deep learning object-detection models used to localize ROIs that contain the tumors in BUS images.

Deep Learning Object-Detection Model	Input Image Size	Architecture	Backbone CNN	Dataset Used for Pre-Training
Faster R-CNN model	640×640	Two-stage object detection	ResNet-50 CNN	MS-COCO
SSD model	640×640	One-stage object detection	ResNet-50 CNN	MS-COCO
EfficientDet-D0 model	512×512	One-stage object detection	EfficientNet-B0 CNN	MS-COCO
CenterNet model	512×512	One-stage object detection	ResNet-50 CNN	MS-COCO

**Table 2:** The ROI detection rates and the mean ± standard deviation values of the precision, recall, and F1-score obtained using the Faster R-CNN, SSD, EfficientDet-D0, and CenterNet object-detection models as well as the proposed edge-based selection method based on the UJH dataset.

ROI Localization Technique	Type of BUS Images	ROI Detection Rate	Precision	Recall	F1-Score
Faster R-CNN model	Benign images	84%	0.86 ± 0.19	0.85 ± 0.10	0.83 ± 0.16
	Malignant images	77%	0.85 ± 0.18	0.84 ± 0.11	0.82 ± 0.14
	All images	81%	0.85 ± 0.19	0.85 ± 0.11	0.83 ± 0.15
SSD model	Benign images	89%	0.86 ± 0.17	0.84 ± 0.13	0.84 ± 0.14
	Malignant images	84%	0.82 ± 0.21	0.83 ± 0.12	0.80 ± 0.16
	All images	87%	0.84 ± 0.19	0.84 ± 0.12	0.82 ± 0.15
EfficientDet-D0 model	Benign images	91%	0.88 ± 0.13	0.86 ± 0.11	0.86 ± 0.10
	Malignant images	86%	0.86 ± 0.15	0.84 ± 0.15	0.83 ± 0.13
	All images	89%	0.87 ± 0.14	0.85 ± 0.12	0.85 ± 0.11
CenterNet model	Benign images	86%	0.89 ± 0.14	0.88 ± 0.10	0.88 ± 0.10
	Malignant images	78%	0.87 ± 0.15	0.86 ± 0.16	0.85 ± 0.13
	All images	83%	0.88 ± 0.15	0.87 ± 0.13	0.86 ± 0.12
The proposed edge-based selection method	Benign images	98%	0.92 ± 0.13	0.91 ± 0.13	0.91 ± 0.13
	Malignant images	97%	0.89 ± 0.13	0.89 ± 0.12	0.89 ± 0.11
	All images	98%	0.91 ± 0.13	0.90 ± 0.13	0.90 ± 0.12

**Table 3:** Comparison between the ROI detection rates and the mean ± standard deviation precision, recall, and F1-score values obtained by the average-, union-, and intersection-based combination methods and the proposed edge-based selection method based on the UJH dataset.

ROI Localization Technique	Type of BUS Images	ROI Detection Rate	Precision	Recall	F1-Score
The average-based combining method	Benign images	97%	0.87 ± 0.18	0.87 ± 0.14	0.86 ± 0.16
	Malignant images	96%	0.85 ± 0.14	0.85 ± 0.12	0.85 ± 0.11
	All images	97%	0.87 ± 0.16	0.86 ± 0.13	0.85 ± 0.14
The union-based combining method	Benign images	98%	0.81 ± 0.16	0.92 ± 0.12	0.85 ± 0.15
	Malignant images	97%	0.77 ± 0.20	0.90 ± 0.15	0.81 ± 0.18
	All images	98%	0.79 ± 0.18	0.91 ± 0.14	0.83 ± 0.16
The intersection-based combining method	Benign images	96%	0.98 ± 0.05	0.70 ± 0.15	0.80 ± 0.13
	Malignant images	94%	0.96 ± 0.11	0.69 ± 0.19	0.79 ± 0.16
	All images	96%	0.97 ± 0.08	0.70 ± 0.17	0.80 ± 0.14
The proposed edge-based selection method	Benign images	98%	0.92 ± 0.13	0.91 ± 0.13	0.91 ± 0.13
	Malignant images	97%	0.89 ± 0.13	0.89 ± 0.12	0.89 ± 0.11
	All images	98%	0.91 ± 0.13	0.90 ± 0.13	0.90 ± 0.12

**Table 4:** The ROI detection rates and the mean ± standard deviation values of the precision, recall, and F1-score obtained using the Faster R-CNN, SSD, EfficientDet-D0, and CenterNet object-detection models as well as the proposed edge-based selection method based on the BUSI dataset.

ROI Localization Technique	Type of BUS Images	ROI Detection Rate	Precision	Recall	F1-Score
Faster R-CNN model	Benign images	76%	0.84 ± 0.23	0.84 ± 0.17	0.81 ± 0.21
	Malignant images	71%	0.83 ± 0.24	0.83 ± 0.20	0.79 ± 0.22
	All images	74%	0.84 ± 0.23	0.84 ± 0.18	0.81 ± 0.21
SSD model	Benign images	85%	0.83 ± 0.19	0.82 ± 0.17	0.80 ± 0.18
	Malignant images	81%	0.81 ± 0.26	0.81 ± 0.19	0.78 ± 0.21
	All images	84%	0.82 ± 0.21	0.82 ± 0.17	0.79 ± 0.19
EffificientDet model	Benign images	87%	0.86 ± 0.12	0.85 ± 0.14	0.84 ± 0.13
	Malignant images	83%	0.84 ± 0.17	0.83 ± 0.12	0.82 ± 0.13
	All images	86%	0.85 ± 0.13	0.84 ± 0.13	0.84 ± 0.13
CenterNet model	Benign images	83%	0.88 ± 0.18	0.87 ± 0.17	0.85 ± 0.18
	Malignant images	77%	0.85 ± 0.19	0.84 ± 0.16	0.83 ± 0.16
	All images	81%	0.87 ± 0.18	0.86 ± 0.17	0.85 ± 0.17
The proposed edge-based selection method	Benign images	99%	0.91 ± 0.15	0.90 ± 0.14	0.89 ± 0.15
	Malignant images	96%	0.87 ± 0.18	0.88 ± 0.15	0.86 ± 0.16
	All images	98%	0.90 ± 0.16	0.89 ± 0.15	0.88 ± 0.16

## Data Availability

The BUSI dataset is available through reference [38].
